# Comparison of bone-to-implant contact and bone volume around implants placed with or without site preparation: a histomorphometric study in rabbits

**DOI:** 10.1038/s41598-020-69455-4

**Published:** 2020-07-24

**Authors:** Merav Folkman, Alina Becker, Isabelle Meinster, Mahmoud Masri, Zeev Ormianer

**Affiliations:** Department of Oral Rehabilitation, The Maurice and Gabriela Goldschleger School of Dental Medicine, Tel Aviv University, Ramat Aviv, 69978 Tel Aviv, Israel

**Keywords:** Medical research, Preclinical research

## Abstract

The objective of this in vivo study was to compare bone-to-implant contact (BIC) and bone area fraction occupancy (BAFO) values of a new implant, designed to be inserted without bone preparation, using two different preparation protocols: no site preparation and prior limited cortical perforation, versus the values of a control implant using a conventional drilling protocol. Forty-one implants were inserted in 13 rabbits. Thirteen test implants with a new thread design were inserted using no bone preparation (NP), 14 test implants were inserted with limited cortical perforation (CP), and 14 conventional implants served as control. Five animals were sacrificed after 21 days and eight animals after 42 days. Histomorphometric analysis was performed and percentage of BIC and BAFO values were measured. ANOVA with Tukey post hoc and Mann–Whitney nonparametric tests were calculated to compare between the groups. Statistical analysis showed no significant difference in the measured values between any of the groups, neither compered by implant nor by compered day. The results demonstrated that biological osseointegration parameters of implant that was inserted without any bone preparation was non-inferior compared to conventional preparation. The clinical relevance is that novel implant designs may not require bone preparation prior to placement.

## Introduction

Various factors influence the long-term prognosis of dental implants and can affect osseointegration, such as surgical technique, host bed, implant surface, implant design, material biocompatibility, and loading conditions^1^. Osseointegration is defined as a direct contact between living bone and the implant on light microscopic level^1^.

A wider definition considers bone quality, as well as stable support of a prosthesis and lack of motion of the implant under functional loads, apposition of new bone, that is identified as normal bone and marrow at microscopic levels, and in direct contact with the implant, without interposed connective tissue^2,3,4^.

Bone-to-implant contact (BIC) percentages is considered essential requisite for implant stability and an indication of successful osseointegration^5,6^.

The most common implant insertion technique is based on a conventional drilling technique, in which gradual expansion of the osteotomy site by sequential enlargement of the drill diameter is performed^8–10^. Conversely, under-preparation of an implant site (also referred to as under-drilling) is defined as preparing the implant’s bed narrower than the implant’s inserted diameter^11–13^ while over-preparation osteotomy refers to preparing an implant’s bed wider than the implant's inserted diameter^13^. Other available techniques for implant insertion are bone compaction, osteodistraction and piezo surgery^8^.

Implant placement without any site preparation is rarely mentioned in published literature; the exception is with regard to orthodontic mini implants which are self-drilling i.e., the implant is inserted without need of predrilling, or "drill free" placement^14^. However, these orthodontic implants are not designated to achieve osseointegration, thus there are no available data regarding BIC and bone area fraction occupancy (BAFO) outcomes for implants placed without any site preparation.

There is disagreement within published literature regarding the influence of under-drilling on osseointegration, specifically on how this technique impacts the BIC and BAFO values. Reported BIC values when using standard drilling techniques as well as in the under-drilling approach range widely from 20.0% to 62.6%, respectively, in various studies^13,15–22^. Most studies reported no statistical difference between the two surgical approaches^15–18,23^, while others report significantly higher BIC values obtained with under-drilling^14–16^ and one study observed significantly lower BIC values in the undersized group^22^.

Implant insertion using the undersized drilling technique results in high insertion torque and consequently higher initial stability^7^. In this press-fit situation, the implant is situated in intimate contact with the bone walls^15^, hence it is more suitable for placement in Type 4 porous bone^23^. However, the undersized technique induces high pressure on the bone walls, mainly on high density bones, which may result in plastic deformation and microcracks.

Most implant's macrostructure is designed to be inserted following bone preparation. In this study, an implant with a unique macrostructure designed to be inserted without any implant osteotomy preparation is presented. The implant was placed following a superficial marking drill with or without preliminary cortical perforation.

The objective of this in vivo animal model study was to measure and compare BIC and BAFO values of a new implant using two different preparation protocols (no site preparation and prior limited cortical perforation), and the values of a conventional implant using a control protocol (conventional drilling technique).

## Methods and materials

**Figure 1:**
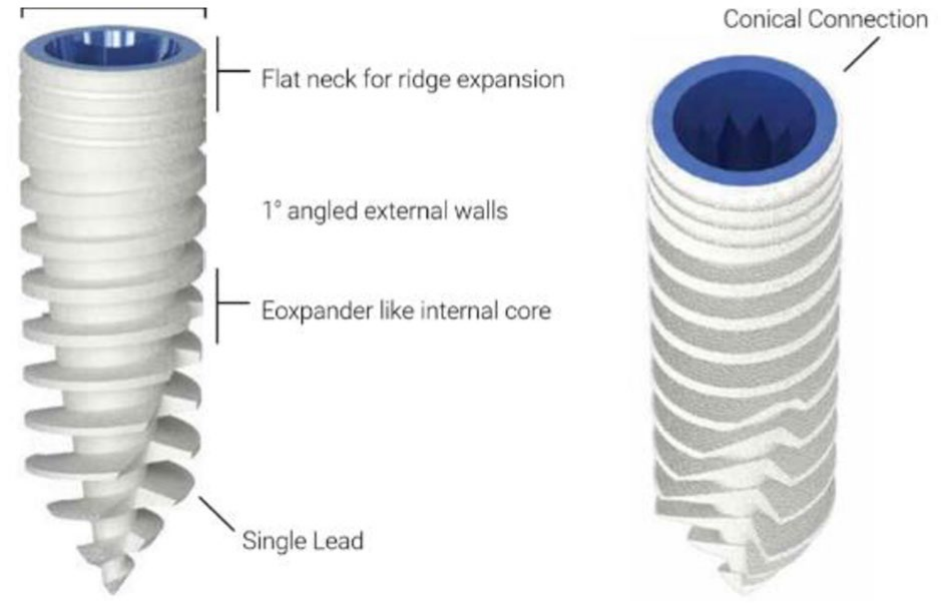
The test implant was a tapered, single lead, self-drilling, conical connection device.

The study protocol was approved by the Research Ethics Committee of the University of Apollonia, Iasi, Romania. A test implant with a new thread design (Magix, Cortex, Israel) (Fig. 1) was inserted using no bone preparation (NP) and cortical perforation (CP) into 13 New Zealand rabbits. The features of the test implants macrostructure enabled insertion without bone preparation due to the single lead threaded tapered design with an expander-like internal core and flat neck, allowing ridge expansion (Fig. 1).

**Figure 2:**
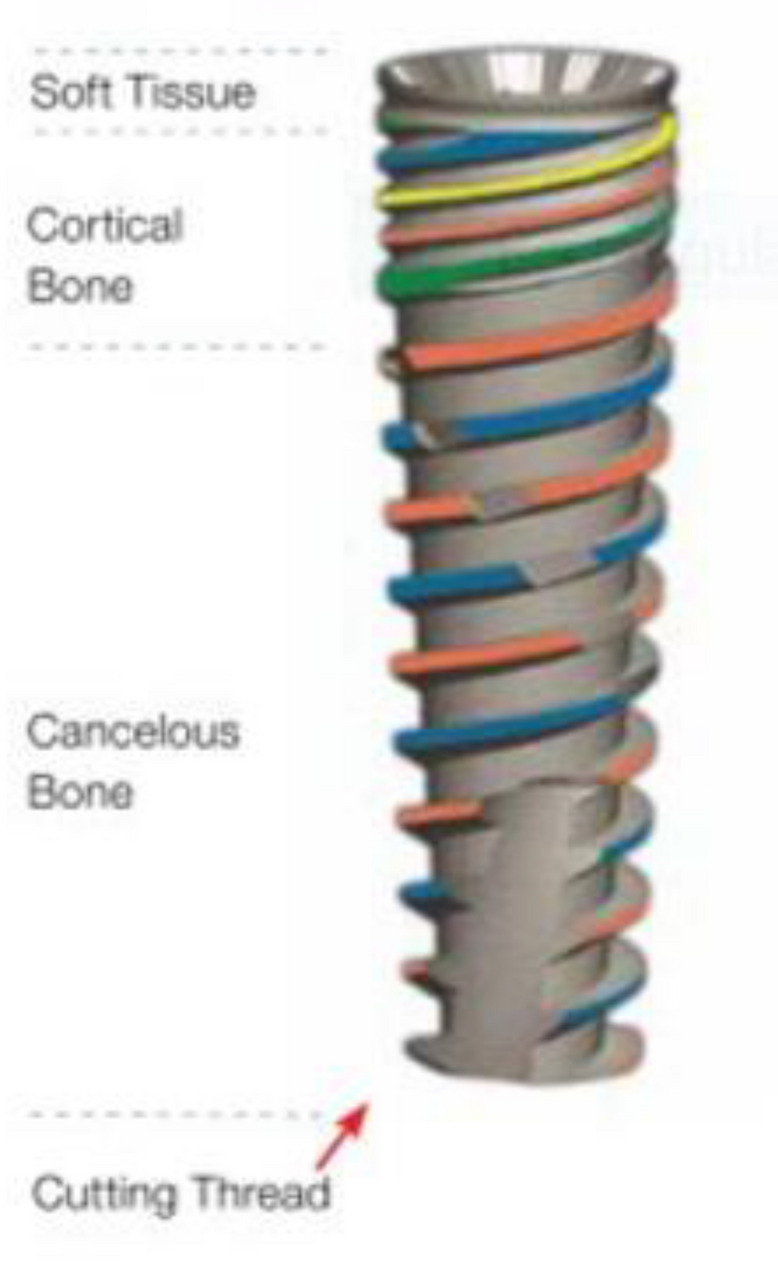
The control implant was a threaded, tapered, double lead, internal hexagonal connection device.

Additionally, a control implant designed for step osteotomy preparation (Dynamix, Cortex, Israel) was also inserted (Fig. 2). Both implants are self-tapping, self-drilling and self-condensing devices made of titanium alloy (Ti6Al4V) with sandblasted acid etched (SLA) surface and share the same dimensions of 10 mm length and 3.8 mm neck diameter.

### Surgical procedure

The animals were anaesthetized using intramuscular injection of a combination of ketamine (Ketamidor 100 mg/ml, Richter Pharma ag, Wels, Bioveta, Czech Republic) (0.25 mg/kg of body weight) and Mepivastesin 3%( 3 M Espe, Germany). Incisions of 5 cm long were performed to expose the surface of the right and left tibial metaphysis. One or two of each type of implant were placed on each side.

#### Implant insertion

**Figure 3:**
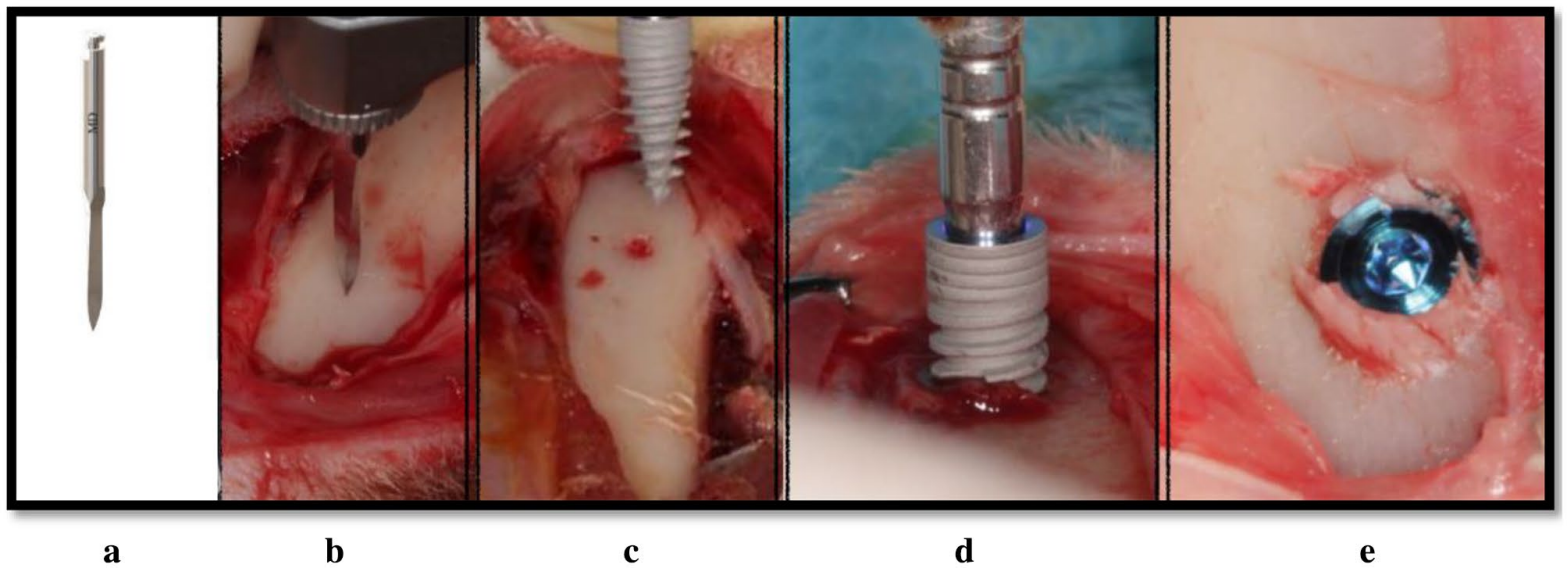
Test group NP surgical procedure in a rabbit’s tibia. **(a)** A marking drill, **(b)** a marking point performed in the bone, **(c)** test implant is used for direct insertion, **(d)** direct placement of the test implant, **(e)** final device position.

**Figure 4:**
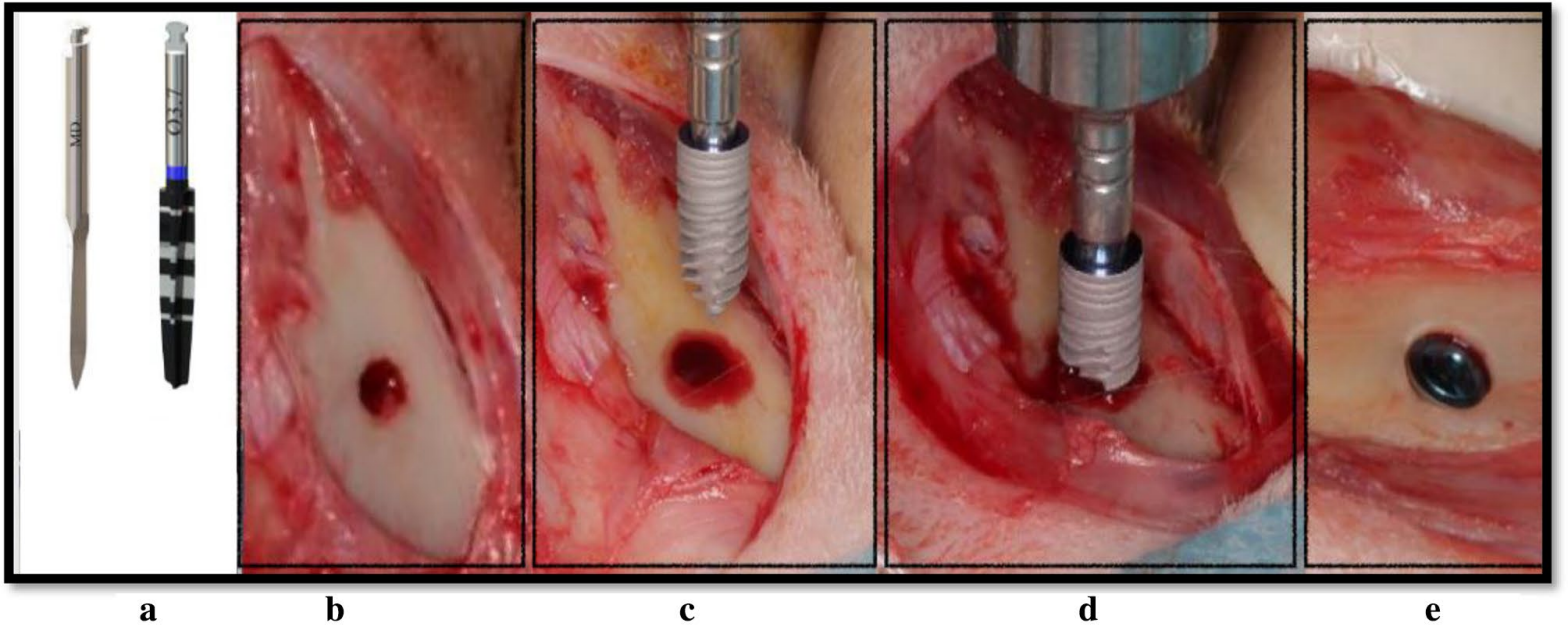
Test group CP surgical procedure in a rabbit’s tibia. **(a)** A marking drill and 3.7 mm conical drill, **(b)** cortical perforation, **(c)**  test implant is used for direct insertion, **(d)**  direct placement of the test implant, **(e)**  final device position.

Three groups were tested and evaluated; all three groups were tested in each of the 13 animals. In group NP, a 1.5 mm bi-cortical marking drill was used followed by direct placement of the test implant (Fig. 3). In group CP, a bi-cortical 1.5 mm marking drill was used, then a 2 mm wide and 1 mm deep cortical bone perforation was performed followed by implant insertion (Fig. 4). In the control group, the implant was inserted according to the manufacturer’s protocol using a transosseous progressive sequence of drills: a marking drill, followed by a 2.0 mm pilot drill, a 2.8 mm drill and a final 3.2 mm drill. A 3.8 mm control implant was inserted under saline cooling.

The soft issues were sutured in separate layers and the animals received postoperatively subcutaneous enrofloxacin 20 mg/kg for 3 days and 0.6 mg/kg subcutaneous meloxicam for 2 days. Five animals were euthanized after 21 days, examining early healing stages of the bone, and 8 animals were euthanized after 42 days representing mature bone formation. Euthanasia was performed by an embutramide product T61 manufactured by MSD. Histology sections of bone samples were taken.

#### Histology and histomorphometry

The bone specimens were infiltrated with Remacryl resin starting with 50% ethanol/resin solution and subsequently 100% resin, with each step lasting 24 h. The photopolymerization was obtained using a 48-h exposure to blue-light. The implants were oriented in order to visualize the two different sides at the same time. After polymerization, the blocks were ground down in order to remove the excess of resin and expose the implant, which was glued on plastic slides using a methacrylate-based glue.

A Micromet high speed rotating blade microtome (Remet, Bologna, Italy) was used to separate the section from the block thus obtaining a 250 µm thick section. The section was then ground down to about 40 µm using a LS-2 grinding machine (Remet, Bologna, Italy), equipped with water-proof grinding paper. Grinding was followed by section polishing using a polishing paper and a 3-µm polishing cream.

**Figure 5:**
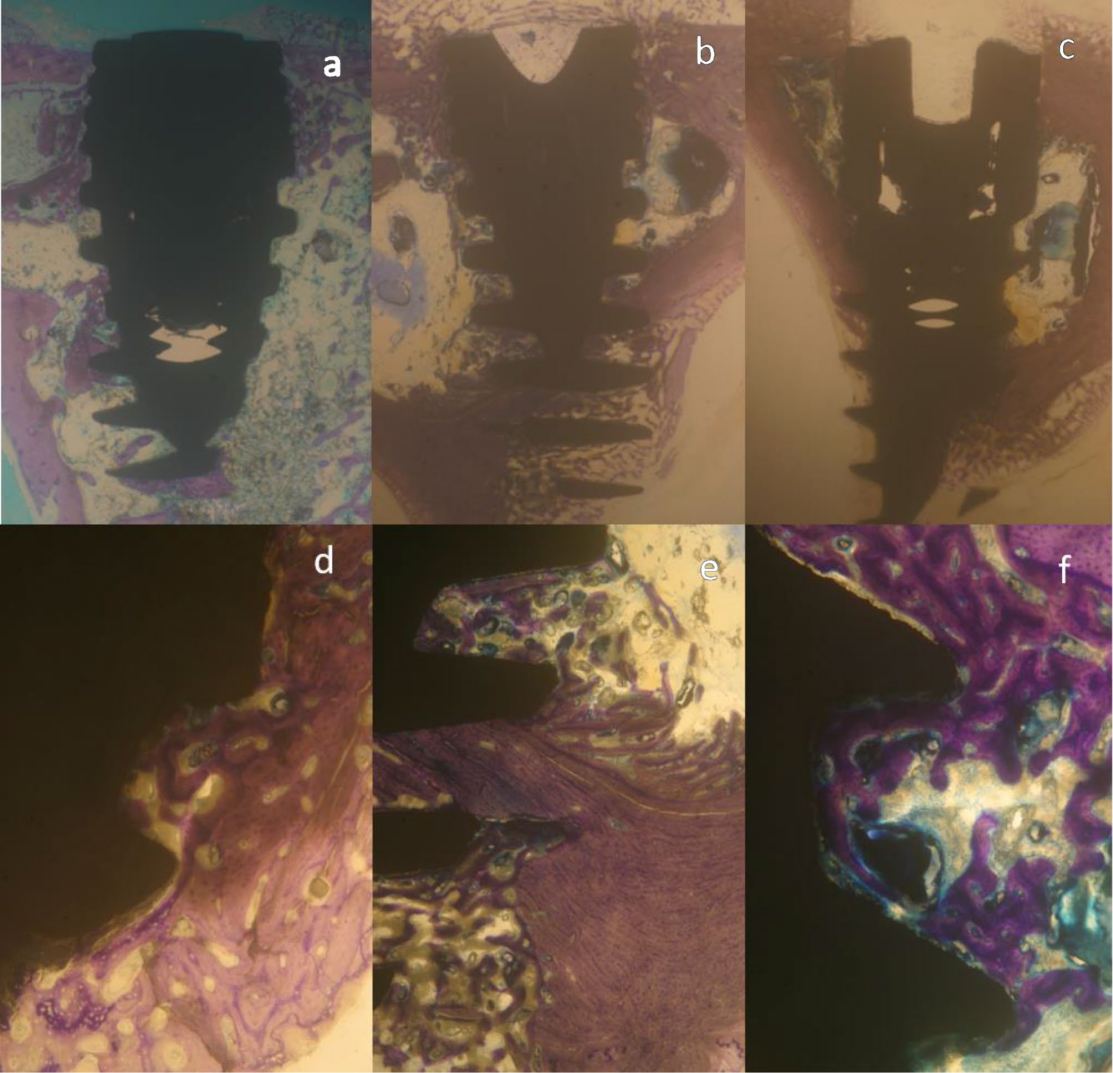
Micrographs of a 21-day site. Representative sections of the implant in × 10 magnification showing the entire implant section at its center, **(a)**  NP group, **(b)**  CP group and **(c)**  control group. Cortical bone is adjacent to the implant neck in all groups, inter-thread space is occupied by bone marrow and newly formed bone. Representative sections of the implant in × 100 magnification showing bone formation at the thread pitch and valley in **(d)**   NP group, **(e)**  CP group and **(f)**  control group. Trabecular bone fills the gaps, osteocytes are evident in all groups.

**Figure 6:**
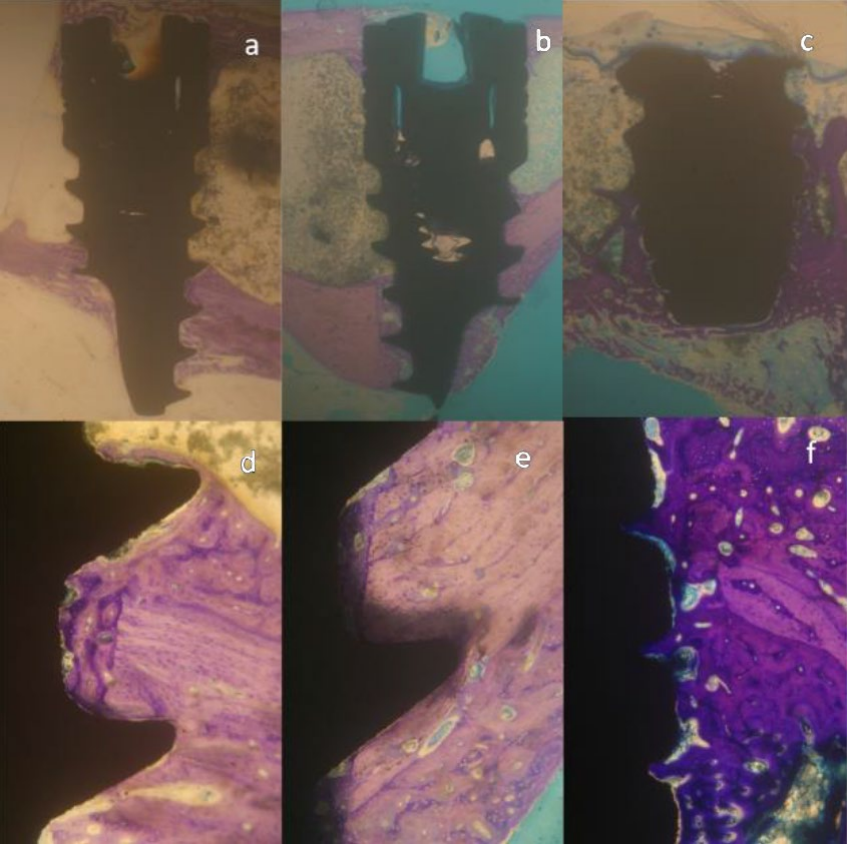
Micrographs of a 42-day site. Representative sections of the implant in × 10 magnification showing the entire implant section at its center, **(a)**  NP group, **(b)**  CP group and **(c)**  control group. The implants are in close contact with the cortical portion of the tibial bone at the neck and apex. The median portion is adjacent to bone marrow. Representative sections of the implant in × 100 magnification showing bone formation at the thread pitch and valley in **(d)**  NP group, **(e)**  CP group and **(f)**  control group. Mature bone formation is seen at the inter-threads space.

The sections were then toluidine blue-stained and referred to optical microscopy at 10 × and 100 × magnification. The histomorphometric analysis was performed by digitizing the images from the microscope via a JVC TK-C1380 Color Video Camera (JVC Victor Company, Japan) and a frame grabber. The images were first acquired with a 10 × magnification, showing the entire implant surface (Figs. 5a–c and 6a–c). Large 100 ×  magnification images were taken for histological evaluation using the image analysis software IAS 2000 (Delta Sistemi, Italy), (Figs. 5d–f and 6d–f). The central section of each specimen was analyzed, and percentage of total implant-bone interface was calculated.

The calculated parameters were the percentage of BIC and BAFO. BIC indicates the percentage of implant surface in direct contact with the bone over the entire length of the implant. The remaining surface is in contact with the marrow tissue (Figs. 5 and 6). BAFO indicates the area occupied by the mineralized bone matrix between the threads over the entire microscopic field. This was measured by outlining the bone surface area from the total field area between the threads and was expressed as percentage.

#### Statistical methods

ANOVA with Tukey post hoc and Mann–Whitney nonparametric tests (also called Wilcoxon rank-sum test) were calculated to compare the control group with NP and CP groups. The Mann–Whitney test was run to confirm the significance of ANOVA results. Because the sample size did not reach the minimal statistical power of 0.80 for all comparisons, a linear regression test was used to get the target power of 0.80 and α = 0.05, and weight function was used. Analyses were conducted using SPSS.

### Ethical approval

All applicable international, national, and/or institutional guidelines for the care and use of animals were followed. All procedures performed in studies involving animals were in accordance with the ethical standards of the institution or practice at which the studies were conducted. The study protocol was approved by the Research Ethics Committee of the University of Apollonia, Iasi, Romania.

## Results

A total of 41 implants were inserted in 13 rabbits, each rabbit received 3 to 4 implants (1 to 2 from each test group, in both the right and left tibiae). Thirteen test implants were inserted in group NP without bone preparation, 14 test implants were inserted in group CP with prior cortical perforation, and 14 conventional implants served as a control group.

Statistical analysis and histological examinations were conducted on a total of 39 implants: 12 implants in group NP, 13 implants in group CP and 14 in the control group. Two implants were excluded due to bone fracture occurring during the surgical procedure, one in group NP, another in group CP. Bone fracture requiring immobilization was diagnosed on post-operative day 1 in 2 additional cases, both in the control group. These implants were included in the statistical analysis.

**Figure 7:**
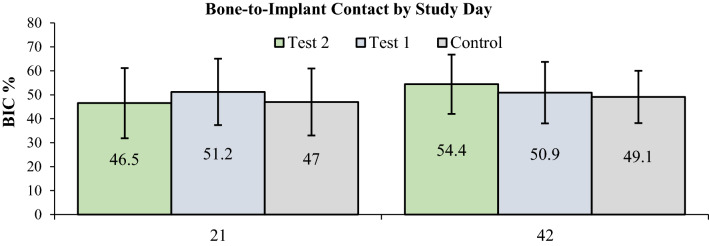
Percentage bone-to-implant contact by study day.

**Figure 8:**
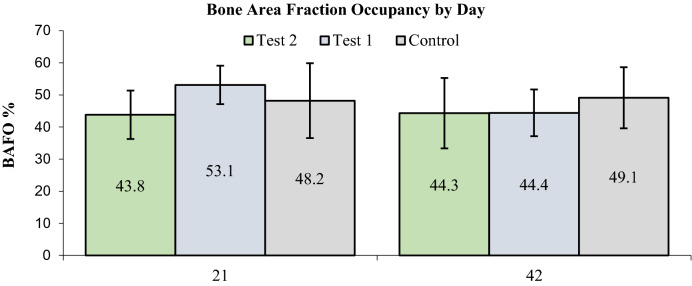
Percentage of bone area fraction occupancy by study day.

There was no statistically significant difference in BIC and BAFO values between group NP and group CP, as well as between the test groups and the control group either on day 21, or on day 42 (Table 1). Histological examination comparing BIC and BAFO by days and by implant type revealed that BIC values ranged from 46.5 to 51.2% on day 21, and from 49.1% to 54.4% on day 42 (p > 0.05). BAFO values ranged from 43.8% to 53.1% on day 21, and from 44.3% to 49.1% on day 42 (p > 0.05), (Figs. 7 and 8). Additionally, no difference was noted within the groups comparing day 21 values to day 42 values (Table 2).

**Table 1:** Bone-to-implant contact and bone area fraction occupancy by study day and implant type.

Study day	Dependent variable	Implant type	N	Mean %	Std. deviation	Observed power	Sample size for power 0.80 α = 0.05 1 side	Weight	p-value*
21	BIC	NP	5	51.2	14.7	0.07	123	12	0.072
		CP	5	46.5	13.9	0.05	8,748	875	0.065
		Control	5	47.0	14.0				
	BAFO	NP	5	53.1	7.6	0.13	41	4	0.055
		CP	5	43.8	6.0	0.11	54	5	0.059
		Control	5	48.2	11.7				
42	BIC	NP	7	50.9	12.4	0.06	497	31	0.070
		CP	8	54.4	12.8	0.14	61	4	0.060
		Control	9	49.1	10.9				
	BAFO	NP	6	44.4	11.0	0.15	47	3	0.084
		CP	8	44.3	7.3	0.18	45	3	0.055
		Control	9	49.1	9.5				

*BIC* bone-to-implant contact, *BAFO* bone area fraction occupancy, *CP* cortical perforation, *NP* no preparation.

*Results adjusted for sample size with power 0.80 and α = 0.05 in one-sided test. The calculated sample size is too small for the approximate calculation performed here to be reliable; hence weight function has been used according to the received sample size.

**Table 2:** Bone-to-implant contact and bone area fraction occupancy means by implant type and study day.

Implant type	Dependent variable	Study day	N	Mean %	Std. deviation	Observed power	Sample size for power 0.80 α = 0.05	Weight	p-value*
NP	BIC	21	5	51.2	14.7	0.05	38,914	3,243	0.070
		42	7	50.9	12.4				
	BAFO	21	5	53.1	7.6	0.27	17	1	0.167
		42	8	44.4	11.0				
CP	BIC	21	5	46.5	13.9	0.16	35	3	0.061
		42	6	54.4	12.8				
	BAFO	21	5	43.8	6.0	0.05	2,749	21 1	0.075
		42	8	44.3	7.3				
Control	BIC	21	5	47.0	14.0	0.06	361	30	0.059
		42	7	49.1	10.9				
	BAFO	21	5	48.2	11.7	0.05	1,395	107	0.070
		42	8	49.1	9.5				

*BIC* bone-to-implant contact, *BAFO* bone area fraction occupancy, *CP* cortical perforation, *NP* no preparation.

*Results adjusted for sample size with power 0.80 and α = 0.05 in one-sided test.

## Discussion

The focus of the current study was to evaluate osseointegration of a new implant designed to be inserted without any prior bone preparation (ie, drill-less) and to compare its biological parameters to a control group that used a conventional implant inserted using a progressive sequence of drills. The study was conducted in an experimental animal model. The results demonstrated that BIC and BAFO values of the test groups were not statistically different compared to the control group, or within the two test groups.

Successful osteointegration relies upon bone formation, which may originate from the implant surface properties: chemical and physical design, this is referred to as “contact osteogenesis”, as well as from the bony margin of the surgical defect i.e. “distance osteogenesis”^2^. In our study, whether the surgical defect was made following direct insertion or after prior preparation did not result in statistical difference in the tested parameters. As to “contact osteogenesis”, chemically wise, both implants have the same surface treatment, however, the physical implant design differs.

Implant design is a key element affecting initial stability and the implant’s ability to sustain load and achieve successful osseointegration^24^. The new implant that was tested has a macrostructure that was specifically designed to be inserted without any initial preparation. It was able to be inserted into the bone in a direct mode, following merely a marking spot or following limited cortical perforation prior to implant placement. Additionally, the features of the test implant’s macrostructure enabled prepless insertion due to the single-lead threaded tapered design with an expander-like internal core and flat neck, allowing for ridge expansion (Fig. 1). The threads of this implant are deep and progressive; thread depth is defined as the distance between the major and minor diameter of the thread, with deeper threads having a positive effect upon implant stabilization in poor bone quality situations. Progressive threads also mean higher depth in the apical portion that decreases gradually coronally. This design is hypothesized to increase the load transfer to the more flexible cancellous bone instead of crestal cortical bone^24^. The test implant features deep and gradual threads (0.3, 0.6, 1.0 mm deep coronally to apically) indicated for insertions in Type 4 bone.

Type 4 bone, present in the human upper jaw, has a very light density, is porous, and presents difficulty when seeking to obtain rigid fixation; as a result, initial stability is often limited. The preparation of Type 4 bone necessitates narrower drills than the dimension of the final implant^25^. This type of preparation is referred to as under—drilling, also known as under preparation or undersized preparation^11^.

The advantages of under-drilling are high insertion torque and initial stability. Other advantages are ease of performance, shortened treatment time, and decreased surgical burden on the patient, as well as minimal postoperative morbidity^9^.

To our knowledge, there is no available data regarding osseointegration parameters in implants inserted without any prior preparation. The influence of under-drilling upon osseointegration is debatable. In a review article that examined the different aspects of undersized implant surgical preparation, the findings regarding BIC values were inconclusive^15^. An in-vivo experimental study evaluated the effect of drilling dimensions upon insertion torque and early implant osseointegration stages. Implants were placed in bone sites using undersized drilling protocol versus a conventional protocol. BIC and BAFO were examined, the results indicated there was no effect of the drilling dimension upon the tested parameters^18^. An additional in vivo experiment compared an undersized drilling protocol versus conventional methods and showed that the BIC percentage calculated was 60.6 ± 14.2% and 47.5 ± 11.7% respectively. The conclusion was that an undersized implant bed can improve BIC^19^.

A different study measured Crestal BIC and Total- BIC, of two implants inserted in an under-drilling or over-drilling protocol, at two time points: 21 or 42 days after insertion. Results demonstrated an increased short-term (21 days) C-BIC, in favor of the over-drilling protocol, however this difference became non-significant statistically at 42 days^26^.

Overall, there are discordant findings in the literature regarding the influence of under-drilling surgical techniques upon biological parameters, however most studies found no difference in BIC and BAFO between tested surgical approaches. The present study’s results demonstrated that BIC and BAFO values of the test groups were not statistically different compared to a control group or within the two test groups. Furthermore, there was no statistical difference within any group comparing BIC and BAFO after 21 days and 42 days. These findings are in agreement with most available data.

Under drilling complications are derived from high insertion torque which may cause bone microfractures and increased temperature generation^27,28^, resulting in compression necrosis of the bone, delayed healing process and increased bone resorption^15,27^. Microfractures occurrence depends on bone density and the discrepancy between the diameter of the implant and the implant’s bed^29^. In the present study two test insertion protocols were conducted. Implant insertion within the first test group was performed without any bone preparation (NP group). In the second test group (CP group), a cortical perforation was performed to avoid compact bone cracks as well as to examine the impact of this difference on the tested parameters. In our work, bone fracture that lead to implant removal occurred in two cases, one in the NP group, another in the CP group. Two other fracture occurrences were diagnosed on the second day in the control group and were followed by immobilization. Because this complication occurred in all three groups and in four different cases, one cannot conclude that using the drill-less implant insertion technique, with or without cortex perforation, affected the outcome.

### Limitations

This study used two different implant types while testing different preparation techniques, which could have confounded our results as these implants differ in their macrostructure. However, the implants in this study have identical surface characteristics; both implants have the same surface treatment, and both are defined as self-tapping, self-drilling, and self-condensing. As mentioned above, it has been established that implant surface properties is an important factor affecting osteoblast differentiation and mineralization by influencing the level of bone-related genes and transcription factors^2,26^.

Another limitation is the sample size which, due to ethical reasons, is relatively small. For approximate calculation performed to be reliable, statistical weight function was used according to the received sample size, in order to achieve statistically validated conclusion.

In conclusion, this study examined and compared the biological parameters of a novel implant, inserted with two different drill-less surgical approaches. Within the limitations of the present study, we can conclude that this technique did not result in an inferior biological outcome as measured (BIC and BAFO) when compared to conventional preparation. Cortical bone perforation prior to implant placement did not affect these factors. Future studies are required to establish more information regarding biomechanical parameters and clinical outcomes when using no or limited bone preparation techniques for implant insertion.
